# Trauma and foreignness: a model of clinical intervention

**DOI:** 10.3402/ejpt.v4i0.22990

**Published:** 2013-10-30

## XIII ESTSS Conference: “Trauma and its clinical pathways: PTSD and beyond”, Bologna, June 2013 ORAL, JUNE 9 HALL LIZ

## Trauma e perdita

### Trauma e stranierità: un modello di intervento clinico   10:45–11:00

#### Chiara Marangio Department of Human Sciences, University of Urbino, Urbino, Italy

Il presente contributo focalizza l'attenzione sulla relazione clinica con soggetti stranieri che riportano quadri post-traumatici. Partendo dalla discussione dei concetti di cultura e di contesto tra paradigmi moderno e post-moderno, si propone un modello generale di intervento clinico implicante una forma di setting negoziale fondato sull'estraneità e sulla contingenza. Contesto e cultura non rispondono a categorie universali e circoscritte spaziotemporalmente ma sono il modo con cui il soggetto significa il mondo. Tale disposizione semiotico-clinica rappresenta un criterio di conoscenza delle configurazioni della persona ma anche uno strumento di emersione delle forme post-traumatiche. Dunque, il clinico conosce sia il modo con cui l'altro dà senso alla propria esperienza che la forma che il trauma assume sul suo funzionamento di base. La prospettiva negoziale prende le distanze da un'idea dell'altro come portatore di sofferenza o come vittima e connota il soggetto come costruttore di esperienza, dunque dotato di agentività. Perciò, è possibile intercettare le risorse personali per orientare un intervento basato sulla contingenza che promuova un processo di co-costruzione di senso, prodotto della relazione tra gli attori. Quest'aspetto generativo interviene sulle forme traumatiche del soggetto agente, aprendo ad una prospettiva di sviluppo e di cambiamento. Il presente approccio psicologico tratta la crisi dell'altro non come categorie fenomeniche, psichiatriche e classificazioniste, ma secondo il senso che l'altro dà alla propria esperienza, anche quando disfunzionale alla propria esistenza. In tal modo, l'oggetto della relazione è il processo relazionale stesso ed è il soggetto nella sua complessità, quindi non solo nella sua accezione di traumatizzato e/o di straniero, condizioni parziali e di stato e non esistenziali.

**Bibliografia**

Carli, R., Paniccia, R. M. (2003). Analysis of the demand. Theory and Technique intervention in clinical psychology. Il Mulino Edition.

Salvatore, S. (2004). Unconscious and speech. The unconscious as a discourse. In M. B. Ligorio (Eds.), *Psychology and Culture: Contexts, Identities and Interventions* (pp. 129–158). Rome, Carlo Amore Edition.

Venuleo, C. (2008). What is the nature of idiographic date? In *Yearbook of Idiographic Science: Vol. 1/2008* (pp. 273–283). Rome, Firera & Liuzzo Publishing.

### Trauma and foreignness: a model of clinical intervention

This paper focuses on the clinical relationship with foreign entities who report the aspects of post-traumatic stress. Starting from the discussion of the concepts of culture and context between modern and post-modern paradigms, a general model of clinical intervention involving a form of negotiation setting based on extraneousness and contingency is proposed. Context and culture do not respond to universal categories and limited space-time frames, but they are the way in which the subject means the world. This semiotic clinical disposal represents a criterion of knowledge of the individual configurations and also an instrument of emerging post-traumatic forms. The clinician knows the way in which the individual gives meaning to their experience and the form that the trauma takes on its basic operation. The negotiating prospect moves away from the idea of the person as a suffering carrier or a victim and connotes the person as a builder of experience, so with agency. It is possible to intercept the personal resources to orient a contingency-based intervention that promotes a process of co-construction of meaning, a product of the relationship between the actors. This generative aspect acts on the traumatic forms of the agent, opening a prospect of development and change. This psychological approach to the crisis is not like the phenomenal, psychiatric and ordinal categories, but in the sense that the subject gives the experience, even when it is dysfunctional for their existence. The object of the relation are the relational process and the person in its complexity, not only in the sense of traumatized and/or a stranger but also as the conditions of partial state and nonexistence.

